# Causas inmunológicas de las manifestaciones bucales de la infección por VIH. Revisión de la literatura

**DOI:** 10.21142/2523-2754-1302-2025-242

**Published:** 2025-05-16

**Authors:** Dayanna Soledad Álvarez Zhapa, Ana Cristina Vásquez Palacios, Magdalena Molina Barahona

**Affiliations:** 1 Carrera de Odontología, Universidad Católica de Cuenca. Cuenca, Ecuador. dayanna.alvarez.14@est.ucacue.edu.ec, rocio.molina@ucacue.edu.ec Universidad Católica de Cuenca Carrera de Odontología Universidad Católica de Cuenca Cuenca Ecuador dayanna.alvarez.14@est.ucacue.edu.ec rocio.molina@ucacue.edu.ec; 2 Especialista en Imagenología Dental y Maxilofacial, Universidad Andrés Bello, sede Concepción. Concepción, Chile. avasquezp@ucacue.edu.ec Universidad Andrés Bello Especialista en Imagenología Dental y Maxilofacial Universidad Andrés Bello Concepción Chile avasquezp@ucacue.edu.ec

**Keywords:** VIH, huésped inmunocomprometido, células (Decs), HIV, immunocompromised host, cells (MeSH)

## Abstract

**Introducción:**

Los pacientes con virus de inmunodeficiencia humana son aquellos que presentan inmunodepresión debido al retrovirus tipo C, el cual puede ocasionar diversas patologías en el sistema estomatológico debido a la deficiencia en la inmunidad que presenta el huésped. Esta inmunosupresión origina que los microorganismos oportunos causen alteraciones dentro de la cavidad oral, lo que causa enfermedades periodontales, candidiasis, sarcoma de Kaposi, etc.

**Metodología:**

Se realizó una revisión bibliográfica integral, basada en la identificación de información relevante disponible entre 2018 y 2024.

**Objetivo:**

El objetivo de la investigación es describir cómo la inmunosupresión del sistema inmunológico del paciente con VIH genera manifestaciones orales.

**Conclusión:**

La inmunodeficiencia causa la pérdida de integridad de la mucosa oral y daño a nivel de los tejidos periodontales. Estas manifestaciones pueden ser ocasionadas por la misma replicación del virus, por medios inflamatorios y la apoptosis de las células inmunitarias.

## Abstract (en)

**Introduction:**

Patients with human immunodeficiency virus experience immunodepression due to type C retrovirus, which can lead to various pathologies in the stomatological system. This occurs due to the immunodeficiency exhibited by the host, resulting from alterations in both systemic and oral cells, thus causing a modified immune response. CD4 T cells, dendritic cells, and macrophages bind to the virus’s DNA and act as sites for viral replication and reservoir of the disease, and undergo apoptosis. Similarly, other cells of the innate immune system become altered. This immunosuppression leads to opportunistic microorganisms attacking opportunely, altering the oral cavity and presenting periodontal diseases, candidiasis, Kaposi's sarcoma, and others.

**Methodology:**

A literature review was conducted to identify relevant information available between 2018 and 2024. Objective: The research aims to describe how immunosuppression of the immune system of a patient with HIV generates oral manifestations.

**Conclusion:**

Immunodeficiency occurring in secondary lines of defense is usually the cause of the loss of oral mucosa integrity, barriers such as immunoglobulins, and damage to periodontal tissues or at the epithelial level. These can be caused by virus replication, inflammatory media, and the apoptosis of immune cells.

## INTRODUCCIÓN

El virus de la inmunodeficiencia humana (VIH) es una infección que tuvo una gran tasa de mortalidad y llegó a ser una de las enfermedades catastróficas en los años 80, cuando surgió en Estados Unidos. Aún sigue siendo un problema de salud pública, ya que es una enfermedad de transmisión sexual (ETS) con mayor relevancia en países de medios y bajos recursos, así como en aquellos que se encuentran en vías de desarrollo o en pobreza extrema ^1^. Según el sesgo epidemiológico del Ministerio de Salud Pública del Ecuador, se estima que en 2021 surgieron 3960 casos nuevos de personas detectadas con VIH; sin embargo, no existen datos o estudios exactos que indiquen posibles lesiones o manifestaciones bucales causadas por el VIH ^2^.

Debemos saber que el síndrome de inmunodeficiencia humana es causado por un retrovirus de tipo C, conocido como lentivirus, el cual interactúa a nivel sistémico y produce una disminución de células CD4, células T, células progenitoras de la médula ósea y timocitos en desarrollo. Ataca las células diana, que son todas aquellas que tienen un receptor específico que va a hormonas, antígenos, anticuerpos, etc., lo que origina una disfunción inmunitaria para el huésped ^3^. 

Se estima que, en el año 2020, a nivel mundial, 37,7 millones de personas se contagiaron con este virus. Por tal motivo, es de suma importancia considerar las formas de transmisión. Los mecanismos de contagio son aquellos que incluyen fluidos corporales o sangre contaminada con el virus ^4^. Se han detectado formas de transmisión del VIH subclasificadas en comunes e inusuales, cuyo detalle puede observase en la Tabla 1 ^5^.

**Tabla 1:** Formas de transmisión del VIH

Comunes	Inusuales
Relaciones sexuales vaginales	Relaciones sexuales orales
Relaciones sexuales anales	Alimentos premasticados
Transmisión perinatal	Besos profundos de boca abierta
Compartir elementos inyectables (agujas, jeringas, calentadores)	Mordeduras
Tatuajes o perforaciones	Transfusión sanguínea o donación de órganos

A pesar de esto, en la actualidad se le considera una enfermedad crónica manejable, puesto que existe la terapia antirretroviral combinada, la cual consiste en la supresión y duración de la réplica vírica plasmática ^6^^-^^8^. Por tal motivo, el objeto de esta investigación es revisar la literatura actual acerca de las causas inmunológicas que provocan manifestaciones bucales en la infección por VIH.

## MATERIALES Y MÉTODOS

### Búsqueda bibliográfica

Se realizó una revisión bibliográfica integral, basada en la identificación de información relevante disponible entre 2018 y 2024, con la intención de indagar el tema de las causas inmunológicas asociadas con las manifestaciones bucales en los pacientes portadores de VIH. De esta manera se recolectó evidencia científica ya existente, para llevar a cabo un manejo eficiente de la condición sistémica y sus implicaciones en el sistema estomatognático. Por tal motivo, dentro del comité de ética se le considero una evaluación exenta, ya que se le considera un estudio sin riesgos.

Para la recolección de datos se implementó una búsqueda exhaustiva por medio de buscadores bibliográficos con bases de datos como PubMed, Scopus, Taylor & Francis Online, mediante palabras claves como VIH, huésped inmunocomprometido, células, citocinas, macrófagos, inmunidad, alteraciones, boca (DeCS).

### Selección de estudios

**Figura 1:**
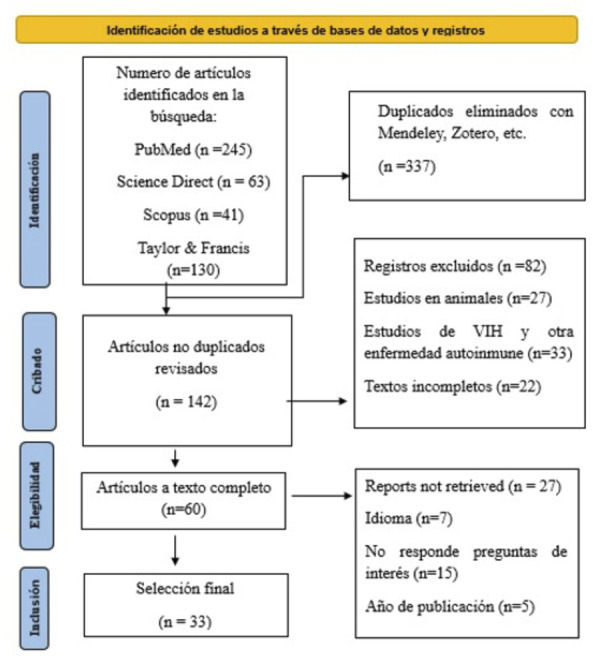
Diagrama de flujo de la búsqueda PRISMA

Para seleccionar artículos relevantes a este tema, se tuvo como criterios de inclusión todos aquellos artículos disponibles y publicados en español, inglés y portugués, según el límite de la búsqueda, que fue desde 2018 hasta 2024. Además, se incluyeron artículos basados en investigación, artículos completos y estudios realizados en humanos. Como criterios de exclusión se consideró artículos publicados antes de 2018, artículos de relevancia científica enfocada en el sida, así como casos clínicos que pueden verse comprometidos con otro tipo de enfermedad sistémica que genere inmunosupresión y ocasione una distorsión. Se excluyó igualmente todo artículo incompleto. Se elaboró una base de datos con el programa Excel 2016, en donde se incluyeron artículos según nuestros criterios de inclusión y exclusión que generan el flujograma PRISMA 2020 (Figura 1).

### Estado del arte

Los pacientes con VIH pueden presentar ciertas manifestaciones principales debido a su inmunodeficiencia. Se ha establecido que para conocer el nivel de supresión inmunológica se emplea el conteo de linfocitos CD4, mientras que para conocer el avance de la infección se observan la carga viral del paciente ^1^. Cuando el recuento de linfocitos CD4 es menor de 500 células por milímetro cúbico en personas con VIH, es posible que comience la aparición de manifestaciones bucales. La cavidad oral pueda ser una zona que genere más importancia dado lo siguiente:

- a. Es un sitio de fácil abordaje para los profesionales.
- b. Es la primera zona donde se expone las manifestaciones de la infección.
- c. Es un indicador de síndrome de inmunodeficiencia adquirida (sida) o avance de la enfermedad, debido a que patologías se presentan por la inmunosupresión avanzada como el sarcoma de Kaposi.
- d. Se observa si la terapia antirretroviral tiene éxito o fracaso por la aparición de manifestaciones.

Las TCD4+ son las primeras células de la segunda línea de defensa, que inhiben la expresión del gen viral; por tal motivo, son consideradas el principal objetivo del VIH. Sin embargo, se sabe que no se necesita únicamente de las CD4 para que el virus se mantenga latente dentro del huésped. A nivel del sistema inmunológico innato, existen diversas células o respuestas inmunitarias que buscan el mismo objetivo que las células linfocíticas, pero que pueden verse afectadas por diversos mecanismos provocados por el VIH o por el propio organismo a nivel sistémico, como se puede observar en la Tabla 2. También pueden generar susceptibilidad en las células de defensa de la cavidad oral, como se aprecia en la tabla 3, lo que da origen a patologías, lesiones y destrucción a nivel de la cavidad oral ^9^^,^^10^. 

**Tabla 2:** Causas inmunológicas en células del sistema inmunológico

Autor y referencia	Año	Causa inmunológica a nivel sistémico	Acción
Chen (^10^)	2022	Células linfocíticas CD4+	Los linfocitos CD4 son los principales reservorios de VIH latente, los cuales en sus células T de memoria de células madre presentan los reservorios de quimiocinas CC5 y CXC4 que son correceptores para la susceptibilidad de la enfermedad, lo que contribuye a la replicación viral y genera un efecto citopático.
Sánchez-Martínez (^11^)	2019	Células linfocíticas CD8+	CD8+ son aquellas que se encargan en la apoptosis celular de agentes ajenos cuando presentan una fusión con réplica viral activa y con cargas de antígenos que generan disminución de las células CD4+.
Akiyama (^12^)	2020	Macrófagos	Podemos encontrar que la quimiocina IP-10 producida por los macrófagos con VIH genera la supresión de las células inmunes, como las células natural killer (NK) y las células T CD8+. A su vez, estas células realizan la latencia del VIH en la célula CD4+.
Mensching (^13^)	2022		
Zhao (^14^)	2021	Activación immune innata	Realiza una nueva reproducción liberando un virión con dos nuevas estructuras del ácido ribonucleico (ARN) del VIH y se recubre con una membrana de células. Esta reconoce a la célula diana, ingresa por la membrana y transcribe el ARN y ácido desoxirribonucleico (ADN) viral inyectándose en el núcleo del genoma del huésped.
Kazer (^15^)	2020	Respuesta proinflamatoria de la activación inmune innata	La proinflamación que realiza el sistema inmune innato con el componente del inflasoma genera supresión, ya que eleva el interferón alfa (IFN -α), quimiocina CXCL10 y las interleucinas, lo que ayuda a la replicación viral.
Pedro (^16^)	2019	Transmisión de célula a célula que presentan un reservorio de VIH-1 latente	Cuando las células CD4+ en reposo presentan una infección se vuelven células de reservorio latente de VIH-1, lo que genera la estimulación de quimiocinas CCL19 y CCL21, y citocinas como las interleucinas 4 y 7 (IL-4, IL-7), lo que provoca susceptibilidad a las células CD4+ no infectadas.
Soare (^17^)	2019	Receptores liberados de los tejidos, linfocitos, macrófagos y células dendríticas	El receptor P2X es un subtipo de receptores tipo TOLL (TLR), el cual se encarga del complejo inflamasoma NLRP3, que somete a la producción de citocinas proinflamatorias. Esto libera interleucina 1β, que genera inflamación y, a la vez, muerte celular de los linfocitos llamada piroptosis, lo cual produce la disminución de células T.

**Tabla 3:** Causas inmunológicas a nivel de células bucales

Autor y referencia	Año	Causa inmunológica a nivel de la cavidad bucal	Impacto	Consecuencia a nivel oral
Coker *et al.* (^18^)	2021	Inmunoglobulinas A y G	Desregularización de las células T CD4, lo que genera un cambio dentro de las células B subepiteliales, y reduce las inmunoglobulinas A y G que se encuentran en los fluidos mucosos.	Se altera la función de hemostasia de la saliva, debido a que existe un cambio a nivel de las células epiteliales que hace que estas inmunoglobulinas disminuyan y deja susceptible la mucosa oral.
Li *et al*. (^19^)				
Coker *et al.* (^18^)	2021	Macrófagos y células dendríticas	El butirato, que es un ácido graso de cadena corta segregado por las bacterias del microbioma oral en el huésped inmunosuprimido, genera apoptosis, regulación de citocinas proinflamatorias y modulación en las proteínas de unión intercelular. Además, origina triptófano y quinurenina, lo que daña la encía y el periodonto.	La inflamación que existe cuando las células se encuentran en interacción con el VIH genera a nivel periodontal daño tisular y perdida ósea.
Li *et al.* (^19^)				
Pérez *et al*. (^20^)				
Coker *et al.* (^18^)	2021	Células proinflamatorias TH17	En las células TH17, la quimiocina CCR6 y el péptido hBD2 disminuyen sus niveles durante la infección en pacientes VIH+, lo que causa pérdida de la integridad de la mucosa oral y disbiosis.	Se pierde la integridad de la barrera que tiene la mucosa oral y la hace más susceptible a infecciones fúngicas -como candidiasis bucal-, inflamación crónica y al cáncer bucal.
Chandavarkar (^21^)	2020	Células de Langerhans (LC)	Este tipo son células dianas para la infección por VIH, ya que se encuentran en el epitelio de la mucosa oral a nivel suprabasal. Los antígenos VIH-1, que estimulan las células CD4+ y CD8+ infectados, contribuyen a la infección.	Cuando las células de Langerhans están en la mucosa oral y son afectadas con el virus por su replicación no producen fagocitosis. Además, cuando la encía se encuentra inflamada, la acumulación de placa bacteriana favorece el aumento de células CD1a+, y se encuentra una posible relación entre la periodontitis y la infección por VIH. Cuando existe inmunodeficiencia, la presencia de células T CD8+ citotóxicas genera apoptosis en el epitelio oral, por lo que las LC generan el aumento del liquen plano, puesto que expresan algunos antígenos que son similares a la Ia.
Weinberg *et al.* (^22^)	2020	Células epiteliales	La proteína quinasa activada por mitógenos (MAPK) es una vía de señalización, la cual brinda señales a los receptores en la superficie celular, lo que regula las células diana. La glucoproteína 120 (gp120) del VIH activa esta proteína y genera alteraciones en la unión y adhesión, lo que permite la entrada paracelular de la infección del virus de forma primaria e incita una infección sistémica.	Cuando existe una activación de las quinasas reguladas por señales extracelulares 1/2 (ERK1/2) y MAPK, se produce una anormalidad de la morfología epitelial, puesto que no se crean las uniones epiteliales y se genera difusión paracelular. Si estas células se encuentran en tejidos malignos o premalignos, pueden acelerar el proceso y evolucionar a cáncer bucal.

## DISCUSIÓN 

La infección del virus de inmunodeficiencia humana es una de las enfermedades crónicas que genera inmunosupresión por medio de diversos mecanismos inmunológicos de alta complejidad, debido a que se produce la destrucción de células dianas que afectan directamente la respuesta inmune y adaptativa. 

En 2022, Chen *et al*. ^10^ concluyeron que el VIH puede evadir la respuesta inmune del huésped debido a que genera reservorios latentes dentro de las células protectoras. Powell *et al*. ^23^ establecieron que las principales células afectadas son las T CD4+, ya que estas, en condiciones de reposo, se transcriben en primera instancia de manera inversa en el ADN del VIH. Este ADN se integra en las células CD4 y, posteriormente, se une a los receptores de células CD4 y sus correceptores CCR5 o CXCR4, lo que genera la entrada a las células. Allí se duplica y produce la disminución de células inmunitarias, causante de la inmunosupresión. Zhang *et al*. ^24^ indican que la unión entre la gp120 y las CD4 forman un tipo de reconocimiento para estos correceptores, lo que da origen a un tipo de tropismo para la infección. Se establece que el CXCR4 genera un tropismo X4, el cual va a ser el más perjudicial debido a que es el encargado de la progresión de la infección, lo cual ocasiona el SIDA, dado que avanza con rapidez la disminución de células CD4. Estas mismas células son afectadas patológicamente por el VIH, proceso en el que juega un papel importante la glicoproteína 120 que se encuentra en la envoltura del VIH. Paim *et al.*^25^ demostraron que esta proteína, además de unirse con las células T por medio de sus correctores, ayuda a una sobreexposición de las células T CD4 a proteínas adaptadoras o receptoras para la muerte celular, como el receptor Fas, lo que estimula el factor de necrosis tumoral (TNFα). Jabea Ekabe *et al.*^26^^)^ señalan que, cuando existe una unión de las células CD4 con gp120, se genera un cambio en la fosfatasa CD45 que produce la muerte celular de timocitos y células mononucleares. Además, se unen los receptores de quimiocina CXCR4, lo que deteriora la tirosina fosfatasa y modifica la vía de fosforilación de los linfocitos T.

En la respuesta innata, tanto en células desarrolladas como en desarrollo, se conoce que los inhibidores NKG2A Y KIR impiden la activación de células NK, las cuales son esenciales para la protección de infecciones. A su vez, estas células pueden contribuir a la destrucción de células T CD4+ por medio de la segregación de NKp44, lo que ayuda al aumento de la virulencia. Además, se ha visto que la capacidad citotóxica de las células NK es mayormente reducida en los niños que no presentan la enfermedad ^27^^,^^28^. Los macrófagos y células dendríticas son dos de las células más afectadas aparte de las células CD4. Se conoce que los macrófagos presentan receptores de quimiocinas, los cuales son los mismos correceptores de las células CD4+ que ayudan a la infección. A su vez, estas células se polarizan hacia distintos fenotipos que tienen mayor o menor carga antiviral. También tienden a mediar la respuesta que da el IFN y TLR, lo que origina la indolamina 2,3-dioxigenasa, que se encarga a una mayor producción de citoquinas proinflamatorias como el TNF y la IL-6, que favorecen la inflamación crónica. Por otro lado, las células dendríticas presentan los receptores que ayudan en la unión de VIH-1; estas presentan medios similares que los macrófagos para la inmunosupresión del huésped por VIH ^27^^-^^30^.

Durante la infección, las células T CD4 se agotan preferencialmente en los tejidos y en sangre, lo que genera una mayor carga vírica. Strongin *et al*. ^31^ reveló que la terapia antirretroviral (TAR) repone este tipo de célula, la cual en un tiempo extenso prolonga el perfil inmunosupresor de las células que persisten con el reservorio latente. Aun cuando el paciente esté en TAR puede existir una translocación microbiana, liberándose citoquinas proinflamatorias y células T CD4 infectadas a nivel oral en mayores cantidades, lo cual genera las infecciones relacionadas con la inmunodeficiencia. En 2018, Presti *et al*. ^32^ hallaron que la correlación entre las células CD14, el receptor TLR2 en saliva y TLR e inflamasoma generan la disbiosis que se caracteriza en la microbiota oral. Bhaskaran *et al*. ^33^ señalan que, en las células que expresan la IL-10 de manera excesiva, se puede generar una alteración en los niveles de FOXP3. Esto sugiere que la desregulación de las células T FOXP3+ podría desempeñar un papel en la disfunción inmune de la mucosa de los pacientes con VIH bajo TAR.

Una respuesta inmunitaria deficiente está relacionada con la disminución de las células Th17, las cuales se encargan de la respuesta inflamatoria y de la protección ante infecciones fúngicas a nivel de la cavidad oral ^19^. Pérez Rosero *et al*. ^20^ indican que la actividad inmunitaria de las células Th2, Th17 e IL-17, presentes en los tejidos de la cavidad oral, se relacionan con la progresión de la enfermedad periodontal en pacientes VIH-positivos. Además, se considera que no solamente el tejido periodontal es el afectado, sino toda la mucosa oral, ya que existe una disminución de su integridad y de elementos protectores como las inmunoglobulinas Ig A en saliva. Las células epiteliales se ven afectadas morfológicamente, lo que facilita la colocación de células infectadas y la neoformación de tejidos que puedan ser patológicamente malignos. Es necesario tener presente que la inmunosupresión provoca la aparición de una variedad de cambios morfológicos a nivel oral, ya sea estados inflamatorios crónicos en tejidos de soporte de las piezas dentales o a nivel de la mucosa, con lesiones ulcerosas o infecciones recurrentes por los microorganismos oportunistas, las cuales pueden terminar en cáncer oral ^18^^,^^20^^-^^22^.

## CONCLUSIÓN

La inmunodeficiencia causada por el VIH puede afectar, a su vez, a células de otros sistemas, como las de la cavidad oral, en donde genera una disminución de la respuesta inmunitaria. Este tipo de virus se replica por medio de su ADN en células dianas, afectándolas y formando células infectadas que producen medios inflamatorios, apoptóticos y, de igual manera, latencia en las mismas, lo que lleva a la pérdida de la integridad de la mucosa oral. Las manifestaciones bucales asociadas con la infección por VIH se producen por una disminución de las múltiples líneas de defensa y, en conjunto con microorganismos oportunistas, pueden afectar principalmente los tejidos periodontales o contribuir a la progresión de ciertas patologías de la mucosa oral, como es el caso del liquen plano, o acelerando los procesos de malignidad en las lesiones precancerosas. Esto se debe a la disminución de diversas barreras inmunitarias orales y sistémicas como inmunoglobulinas, neutrófilos, entre otros, lo que es explicado anteriormente en esta revisión.

## Financiamiento:

Autofinanciado
